# Validation of a modified thromboelastometry approach to detect changes in fibrinolytic activity

**DOI:** 10.1186/s12959-016-0076-2

**Published:** 2016-01-14

**Authors:** Gerhardus J. A. J. M. Kuiper, Marie-Claire F. Kleinegris, René van Oerle, Henri M. H. Spronk, Marcus D. Lancé, Hugo ten Cate, Yvonne M. C. Henskens

**Affiliations:** Department of Anaesthesiology and Pain Treatment, Maastricht University Medical Center (MUMC+), P. Debyelaan 25, PO Box 5800, 6202 AZ Maastricht, The Netherlands; Department of Internal Medicine, Cardiovascular Research Institute Maastricht, Laboratory for Clinical Thrombosis and Haemostasis, Maastricht University Medical Center (MUMC+), Maastricht, The Netherlands; Central Diagnostic Laboratory, Cluster for Hemostasis and transfusion, Maastricht University Medical Center (MUMC+), Maastricht, The Netherlands

**Keywords:** Blood coagulation tests, Fibrinolysis, Thrombelastography, Tissue plasminogen activator, Validation studies

## Abstract

**Background:**

Thus far, validated whole blood assays used in in vitro fibrinolysis experiments using thromboelastometry (ROTEM) are lacking or have yet to be tested in humans.

The objective was first, to establish a standardized modified ROTEM approach to detect both hypo- and hyperfibrinolysis. And second, to perform a technical and clinical validation of the assay.

**Methods:**

Blood was used of healthy volunteers, patients with sepsis, patients after cardiothoracic surgery, pregnant women, and cirrhotic liver disease patients. A whole blood tissue factor (TF) activated ROTEM assay with and without the addition of recombinant tissue plasminogen activator (rTPA) was developed. Plasma fibrinolysis determinants were measured in all volunteers and patients.

**Results:**

Thirty five pM TF and additions of 125 and 175 ng/ml rTPA resulted in full lysis within 60 min in healthy volunteers. Coefficients of variation were below 10 % without and below 20 % with rTPA addition. In sepsis the hypofibrinolytic ROTEM profiles with 175 ng/ml rTPA were in line with the plasma determinants (high PAI-1, high fibrinogen, low tPA activity, and high d-dimers). After cardiothoracic surgery, reduced fibrinogen and platelet levels accounted for the reduced maximum clot firmness. The hypofibrinolytic profile is attributed to tranexamic acid use and elevated PAI-1 levels. The lowest rTPA concentration in cirrhosis resulted in hyperfibrinolysis in only few of the patients. In pregnancy normal profiles were found.

**Discussion:**

Our high rTPA concentration demonstrates hypofibrinolytic profiles adequately in sepsis and after cardiothoracic surgery. Our low rTPA concentration of 125 ng/ml seems too high for demonstrating hyperfibrinolysis in cirrhotic liver disease.

**Conclusions:**

We were able to present a validated whole blood ROTEM approach to fibrinolysis testing using added rTPA, which can be of added value next to classical plasma based fibrinolysis assays.

## Background

Fibrinolysis is the process of degradation of a fibrin clot into so-called fibrin degradation products (FDPs) of which the d-dimers are best known. Following coagulation, tissue-type plasminogen activator (tPA) induces fibrinolysis by activation of plasminogen into plasmin which enzymatically degrades fibrin into FDPs. To avoid excess clot lysis activity, the fibrinolytic system is under tight control of alpha 2-antiplasmin, plasminogen activator inhibitor-1 (PAI-1), and thrombin activated fibrinolysis inhibitor (TAFI). However, a disbalance in pro- and antifibrinolytic factors can result in either hyper- or hypofibrinolysis, which may contribute to the risk for bleeding or thrombosis, respectively. In trauma and cirrhosis the increased bleeding tendency is attributed to a hyperfibrinolytic state in combination with a rebalanced hemostatic system [1, 2]. In pregnancy, a hypercoaguable state may occur in conjunction with reduced fibrinolysis [3]. In sepsis, the initial procoagulant state is associated with attenuated fibrinolysis due to elevated PAI-1, which may advance towards an uncontrolled syndrome of disseminated intravascular coagulation (DIC), with a clinical bleeding tendency [4, 5]. Factor XIII (FXIII) activated by thrombin crosslinks fibrin strands in order to strengthen the fibrin clot. Low FXIII activity levels are seen in sepsis and could contribute to an enhanced bleeding diathesis in severe septic shock [6].

One of the therapeutic approaches to diminish overt fibrinolysis is treatment with tranexamic acid, which directly inhibits the action of plasmin. For instance, in cardiothoracic surgery this antifibrinolytic drug is being used to prevent and counteract extracorporeal circulation (ECC) induced hyperfibrinolysis [7], whereas in trauma the drug is used to diminish the bleeding tendency [8].

Both the diagnosis of the fibrinolytic state and the management of therapeutic interventions aimed at the fibrinolytic system could benefit from a sensitive but also robust laboratory assay. Although most proteins and enzymes involved in fibrinolysis can be quantified these do not properly reflect the dynamic status of the fibrinolytic system. Assays developed to detect hypo- or hyperfibrinolysis are not commonly used in daily practice because they are cumbersome, plasma-based and/or not suitable for bedside assessment [9]. Several groups have modified overall hemostatic assays such that they would become more sensitive to changes in the fibrinolytic system. This was achieved by modifying whole blood thrombelastography (TEG) [10] and rotational thromboelastometry (ROTEM) [11] by addition of recombinant TPA (rTPA) to induce fibrinolysis ex vivo. More research has been done using other methods or in specific patient groups [12–21]. Overall, however, these studies lacked clinical validation in healthy volunteers as well as defined patient subsets with known abnormalities in coagulation and/or fibrinolysis.

The goal for our modified ROTEM test was to use whole blood, be tissue factor activated, have one application with and one without the addition of rTPA, and would have similar reproducibility in comparison to the commercial non-modified ROTEM tissue factor activated test (EXTEM). Ideally, it would have short(er) runtimes in comparison to plasma based fibrinolysis assays or markers, have good distinction between healthy volunteers and patient groups, be easy to interpret, and have results in line with plasma based markers for fibrinolysis. In this report we describe the development, the technical, and the clinical validation of a novel whole blood ROTEM based assay application including rTPA to induce fibrinolysis.

## Methods

### Group selection

The institutional Ethics Committee of the Maastricht UMC+ approved this study and of all subjects informed consent was obtained. No compensation was given for participation in this study. Blood of healthy volunteers was used to validate the assay and to define reference intervals, while the blood of 4 patient groups (sepsis, cardiothoracic surgery (CTS), 3^rd^ trimester pregnancy, and cirrhotic liver disease Child-Pugh score B or C) was used for clinical validation. Any notion of anticoagulation use in a subject was reason to exclude this subject from the study. Septic patients were recruited within 1 h after the onset of sepsis as judged by the intensive care doctor in charge according to current guidelines [22] after which prompt blood withdrawal was executed. Only septic patients without signs of (non-)overt DIC were included [23]. Cardiothoracic surgery patients were recruited before surgery. Directly after the surgery upon arrival at the ICU blood withdrawal was done promptly. Patients receiving heparin were antagonized with protamine in a 1:1 ratio before arrival on the ICU according to local protocol. Females in their 3^rd^ trimester of pregnancy were recruited at the policlinic during regular check-ups or at the ward before having elective caesarean section the next day. Child-Pugh score B and C cirrhosis patients were recruited via the policlinic or at the ward after which blood was withdrawn.

### Preparation

Human recombinant tissue factor (Innovin, Dade Behring Marburg GmbH, Marburg, Germany) was reconstituted according to the manufacturer’s instructions obtaining a 1 IU/mL concentration. This TF solution together with CaCl_2_ at 1 M and HN-Buffer (20 mM HEPES, 150 mM NaCl, pH 7.7) was prepared in advance and kept at 4–7 °C till further use.

Recombinant tPA (Actilyse, Boehringer Ingelheim, Alkmaar, the Netherlands) was reconstituted according to the manufacturer’s instructions (1 mg/mL or 580,000 IU/mL alteplase), immediately aliquoted, frozen, and kept at −80 °C till further use.

The blood withdrawal was performed by venipuncture discarding the first 3 mL or, if a radial artery catheter was in situ, by flushing the first 5 mL before the blood was collected in vacuum tubes containing 3.2 % (w/v) citrate (Vacutainer, Becton Dickinson BV, Breda, the Netherlands), 11 mM citrate with 50 μg/mL corn trypsin inhibitor (CTI, Haematologic Technologies Inc., Essex Junction, VT, USA) and 0.5 M citrate at pH 4.3 (TriniLIZE Stabilyte, Tcoag Ireland Limited, Bray, Ireland). Tubes were gently mixed directly after blood withdrawal.

### ROTEM analysis

The ROTEM device and cups and pins were from TEM International GmbH (Munich, Germany). The device temperature was set to 37 °C and the maximum runtime to 120 min. TF, CaCl_2_, and rTPA (if applicable) were added to 300 μL whole blood to obtain final concentrations of 35 pM TF, 10 mM CaCl_2_, and 0, 125, and 175 ng/mL rTPA (which equals 0, 72.5, and 101.5 IU/mL alteplase). Total volume of the reagents was maintained at 50 μL per cup by applying adequate volumes of HN-buffer.

The following standard ROTEM parameters were analyzed per channel: CT (clotting time in seconds), MCF (maximum clot firmness in millimeters), LOT (lysis onset time in minutes), and LT (lysis time in minutes). The following derived parameters were calculated using the ROTEM data: the FS (fibrinolysis speed, defined as the decline in %/min between the LOT and LT points; thus: FS = 75/(LT_min_-LOT_min_)) and the FS_c_ (the corrected fibrinolysis speed in mm/min between the LOT and LT points; thus: FS_c_ = (amplitude_LOT_-amplitude_LT_)/(LT_min_-LOT_min_)).

### Euglobulin clot lysis time analysis (ECLT)

The euglobulin clot lysis time was performed similar to [24]. Differently, the precipitate was dissolved in 1 ml Veronal buffer and the reaction was started by adding 25 μl thrombin 100 U/ml. Every 10 min resolution of the clot was assessed visually for the first two hours. Afterwards it was observed every 15 min up to two hours. If a clot was still present at two hours, a last observation was made at four hours.

### Complete blood count

Haemoglobin, haematocrit, platelet, and white blood cell counts were performed on a Sysmex XE-5000 (Sysmex Nederland B.V., Etten-Leur, The Netherlands) using citrated whole blood. Final values were adjusted for the extra 1/9^th^ volume of citrate present during the measurements.

### Preparation of platelet poor plasma (PPP)

Blood was spun twice to gather PPP: a first step at 2,500 *g* for 5 min, followed by a second step at 10,000 *g* for 10 min. The PPP was directly aliquot, frozen, and kept at −80 °C till further use.

### PT (prothrombin time), aPTT (activated partial thromboplastin time), alpha 2-antiplasmin activity, plasminogen activity, Factor XIII activity, d-dimer level, and fibrinogen level

A BCS® XP System (Siemens Healthcare Diagnostic B.V., The Hague, The Netherlands) was used for determining PT (Dade Innovin), aPTT (Dade actin), alpha 2-antiplasmin activity (Berichrom α2-Antiplasmin), plasminogen activity (Berichrom Plasminogen), factor XIII activity (Berichrom Factor XIII), d-dimer levels (Innovance D-Dimer), and fibrinogen levels (Multifibrin U) in PPP according to the manufacturer’s instructions in batches.

### tPA activity, PAI-1 activity, and TAFI antigen

ELISA kits for measuring tPA activity (ZYMUPHEN tPA Activity), PAI-1 activity (ZYMUTEST PAI-1 Activity), and TAFI antigen (ZYMUTEST proTAFI Ag) were acquired from Hyphen Biomed (Neuville-Sur-Oise, France) and measured in PPP according to the accompanied instructions in batches.

### Statistical analysis

Data were collected and analyzed with IBM SPSS Statistics v19.0.0 (IBM Corporation, Armonk, NY, USA). Categorical data are presented as n (%), continuous data as median and inter quartile range (IQR).

For reproducibility, coefficients of variation (CV’s) are expressed as the ratio of the standard deviation to the mean. Between run CV’s were calculated using 16 repeated measurements with blood of one withdrawal of one healthy volunteer. For 0 ng/mL rTPA CT and MCF values were analyzed and for 125 and 175 ng/mL rTPA LOT and LT values besides the CT and MCF. Furthermore within run CV’s were calculated from 11 different blood withdrawals in one person over a time span of six months. In this case CT, MCF, LOT, and LT were assessed at 175 ng/ml rTPA.

Optimal time from blood withdrawal to measurement was assessed by using blood of three healthy volunteers and keeping it at 37 °C till further use. CT, MCF, LOT, and LT were measured using the third condition (i.e. 175 ng/mL rTPA) at 30 min, 1 h, 2 h, 3 h, and 4 h after blood withdrawal and assessed for stability. For each time point a new vial of CaCl_2_, TF, and rTPA was used.

Ninety five percent reference ranges were determined using MedCalc v12.0® (MedCalc Software, Mariakerke, Belgium) using data from healthy volunteers. Skewed data were normalized first and afterwards transformed back when calculating the 95 % reference ranges. Mann–Whitney-U was used for comparison testing. Determinants of the ROTEM parameters and their 95 % confidence interval (95 % CI) were calculated by multiple linear regression analysis assuming *p* < 0.05 as statistically significant. Figures were constructed using Graphpad Prism v5.0a (GraphPad Software, San Diego, CA, USA).

## Results

### Technical validation

Before establishing the optimal concentrations for the rTPA induced fibrinolysis ROTEM assay, various configurations of TF and rTPA concentrations were investigated. This revealed that TF concentrations below 35 pM resulted in prolonged CT values, whereas the MCF was hardly influenced (data not shown). Therefore, the final TF concentration was set at 35 pM. In blood from healthy volunteers, full clot lysis was obtained at a final rTPA concentration of 125 ng/mL around 60 min, while at a final concentration of 175 ng/mL rTPA full lysis was observed around 45 min (data not shown). These concentrations were appropriate to maintain acceptable short runtimes. At both 125 and 175 ng/mL rTPA, ROTEM was characterized by a lower MCF compared to the assay without tPA (125 ng/mL rTPA: 2 mm (a 3.6 % decrease) and 175 ng/mL rTPA: 5 mm (a 8.4 % decrease)), while the CT prolonged by 5 s (a 11 % increase) and 7 s (a 17 % increase) respectively in comparison to the assay without rTPA.

Assessment of reproducibility for the different ROTEM parameters showed between run CV’s of 9.5 % and 1.8 % for the CT and MCF at the 0 ng/mL rTPA concentration. Between run CV’s were 8.7 %, 4.1 %, 12.1 % and 18.8 % for CT, MCF, LOT, and LT at the 125 ng/mL rTPA concentration and for 175 ng/mL rTPA CV’s were 12.1 %, 4.9 %, 16.6 % and 17.1 % for each parameter, respectively.

Within run CV’s showed 25 % for CT, 8.4 % for MCF, 16.0 % for LOT, and 27.2 % for LT at 175 ng/ml rTPA.

**Fig. 1:**
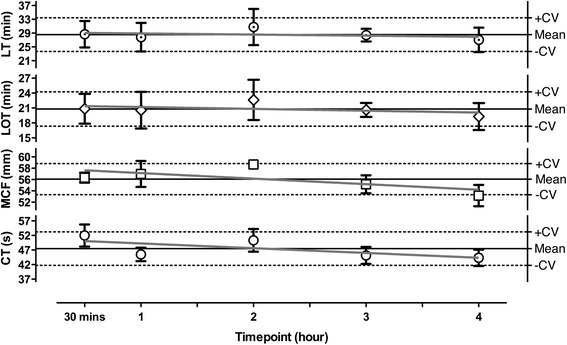
Whole blood stability when performing rTPA induced fibrinolysis on a ROTEM. Whole blood stability at room temperature (at 0,5 to 4 h after blood drawing) of 3 healthy volunteers for ROTEM parameters at 175 ng/mL rTPA; mean per time point (±SEM); overall mean (solid horizontal line on Y-axis) ± error margin based on CV of test (dotted horizontal lines on Y-axis); fitted line through all value points (grey declining line). *CT* clotting time, *CV* correlation of variation, *MCF* maximum clot firmness, *LOT* lysis onset time, *LT* lysis time

To determine whole blood stability in the assay, ROTEM analysis was started at 30, 60, 120, 180, and 240 min after blood drawing. At 175 ng/mL rTPA, all ROTEM derived parameters remained stable up to 4 h after sample collection (Fig. 1). Comparable results were obtained for the assay in the presence of 125 ng/mL rTPA (data not shown).

### Reference intervals

ROTEM analysis data from 40 healthy volunteers (male to female ratio of 1:1) was used to calculate the 95 % reference intervals (Table 1). Age, height, body weight, haemoglobin, and haematocrit level were higher in males compared to females (mean difference: 25 years, 14 cm, 27.5 kg, 1.1 mmol/L, and 0.04 L/L, respectively: all *p* < 0.05) while platelet count, white blood cell count, and fibrinogen levels were not different between the two sexes. Regarding the ROTEM parameters, CT values were significantly different between the two sexes with males having shorter CTs for all rTPA concentrations (6.5, 12.8, and 14.5 s at 0, 125, and 175 ng/mL rTPA respectively: all *p* < 0.05). Differences between males and females were not observed for the other ROTEM parameters: i.e. MCF, LOT, and LT for any of the three conditions.

**Table 1:** 95 % reference intervals for tissue factor stimulated ROTEM parameters in whole blood of 40 healthy volunteers using different concentrations of tPA

	0 ng/mL rTPA	125 ng/mL rTPA	175 ng/mL rTPA
CT (s)	36–72^a^	33–75^a^	29–73^a^
MCF (mm)	52–70^a^	47–68^a^	39–66^a^
LOT (min)	73–120	26–49^a^	18–40^a^
LT (min)	>120	35–77^a^	25–57^a^
FS (%/min)	NA	2.5–9.8^a^	4.1–12.0
FS_c_ (mm/min)	NA	1.4–5.7^a^	2.4–6.6^a^

^a^after normalization and transformation

*rTPA* Recombinant tissue plasminogen activator, *CT* Clotting time, *MCF* Maximum clot firmness, *LOT* Lysis onset time, *LT*, Lysis time, *FS* Fibrinolysis speed, defined as the decline in %/min between the LOT and LT points thus: FS = 75/(LT_min_-LOT_min_), *FS*_c_ Corrected fibrinolysis speed in mm/min between the LOT and LT points thus: FS_c_ = (amplitude_LOT_-amplitude_LT_)/(LT_min_-LOT_min_)

### Clinical validation

In total we included 21 sepsis patients, 15 cirrhosis patients, 7 females in their 3^rd^ trimester of pregnancy, and 20 cardiothoracic surgery (CTS) patients of which one patient did not receive tranexamic acid (this patient was omitted from all statistical analyses).

Complete blood count (Hb, Ht, WBC, and PLT) and measurement of PT and aPTT was performed revealing results that are compatible for these patient groups (Table 2).

**Table 2:** Complete blood count and PT and aPTT of the healthy volunteers and patient groups

	Healthy volunteers	Sepsis	CTS-TXA	Cirrhotic liver disease	Pregnancy
Hb (mmol/L)	9.0 (8.4–9.6)	6.0^*^ (5.6–6.3)	6.2^*^ (5.7–6.7)	7.7^*^ (7.2–8.4)	7.8^*^ (7.0–8.4)
Ht (L/L)	0.43 (0.40–0.45)	0.29^*^ (0.27–0.31)	0.29^*^ (0.26–0.32)	0.38^*^ (0.34–0.39)	0.36^*^ (0.34–0.39)
PLT (x10^9^/L)	178 (139–212)	141 (95–213)	101^*^ (91–140)	99^*^ (62–136)	202 (127–234)
WBC (x10^9^/L)	6.4 (5.4–7.9)	20.1^*^ (11.5–25.3)	10.7^*^ (8.3–12.4)	5.0 (4.0–7.5)	10.7^*^ (9.3–11.7)
PT (s)	10.5 (10.2–10.8)	11.5^*^ (10.7–12.1)	11.8^*^ (11.5–12.4)	13.3^*^ (13.0–13.5)	9.6^*^ (9.3–9.6)
aPTT (s)	27 (26–27)	33.5^*^ (30–42.5)	30^*^ (28–33)	29^*^ (29–34)	25^*^ (23–25)

Results are median (IQR); ^*^ *p* < 0.05 in comparison to healthy volunteers

*Hb* Hemoglobin, *Ht* Hmatocrit, *PLT* Platelet count, *WBC* White blood cell count, *PT* Prothrombin time, *aPTT* Activated partial thromboplastin time *IQR*, Interquartile ranges, *CTS-TXA*, Cardiothoracic surgery patients using tranexamic acid

**Fig. 2:**
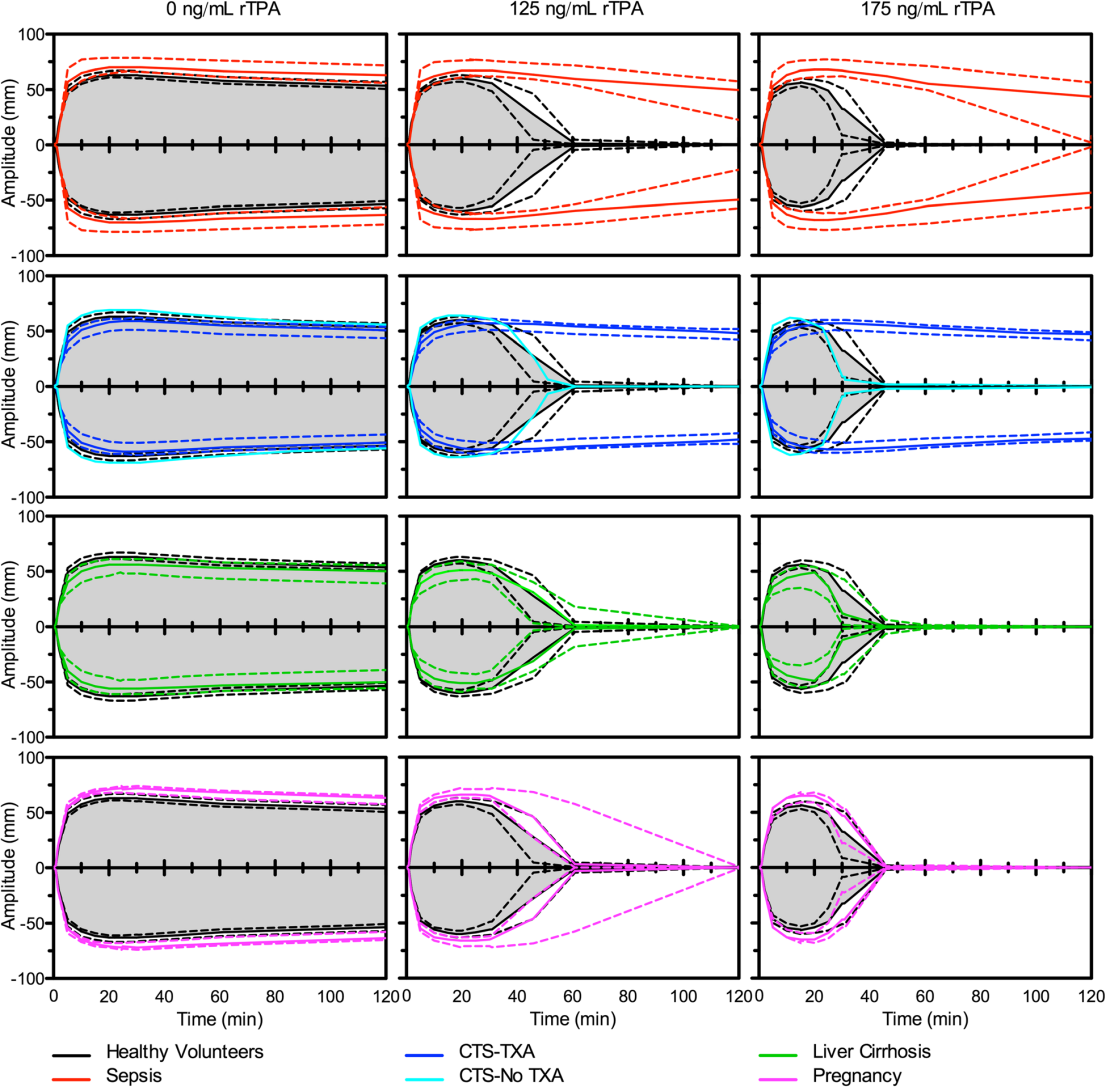
Normal and rTPA induced fibrinolysis profiles on a ROTEM device. Median tissue factor stimulated ROTEM traces (coloured solid lines) with different rTPA concentrations expressed as interquartile ranges (coloured dotted lines) of four patient groups compared to healthy volunteers (black and grey). *CTS-TXA* cardiothoracic surgery patients using tranexamic acid, *CTS-No TXA* cardiothoracic surgery patient not using tranexamic acid, *rTPA* recombinant tissue plasminogen activator

In Fig. 2 a comparison of the ROTEM profiles for each patient group to the group of healthy volunteers is displayed. As an example, the cardiothoracic surgery patient without having had tranexamic acid was added to this graph only.

In the absence of rTPA, the CT was shorter for cirrhosis (*p* = 0.0121), equal for pregnancy (*p* = 0.5289), and longer for sepsis (*p* < 0.0001) and CTS (*p* = 0.0013) in comparison to healthy volunteers. Although all statistically significant, none of the median clotting times of the groups was outside the normal range established in healthy volunteers. The MCF was lower for CTS (*p* = 0.0001) and cirrhosis (*p* = 0.0014), while it was higher in sepsis (*p* = 0.0003) and pregnancy (*p* = 0.0008). The MCF in sepsis and pregnancy were both statistically as well as clinically significant in comparison to the healthy volunteers. LOT was prolonged in sepsis (*p* = 0.0041) and CTS (*p* = 0.0206), but was equal in cirrhosis (*p*= 0.1624) and pregnancy (*p* = 0.1279). The LT could not be determined in any of the groups within the runtime of 120 min.

**Fig. 3:**
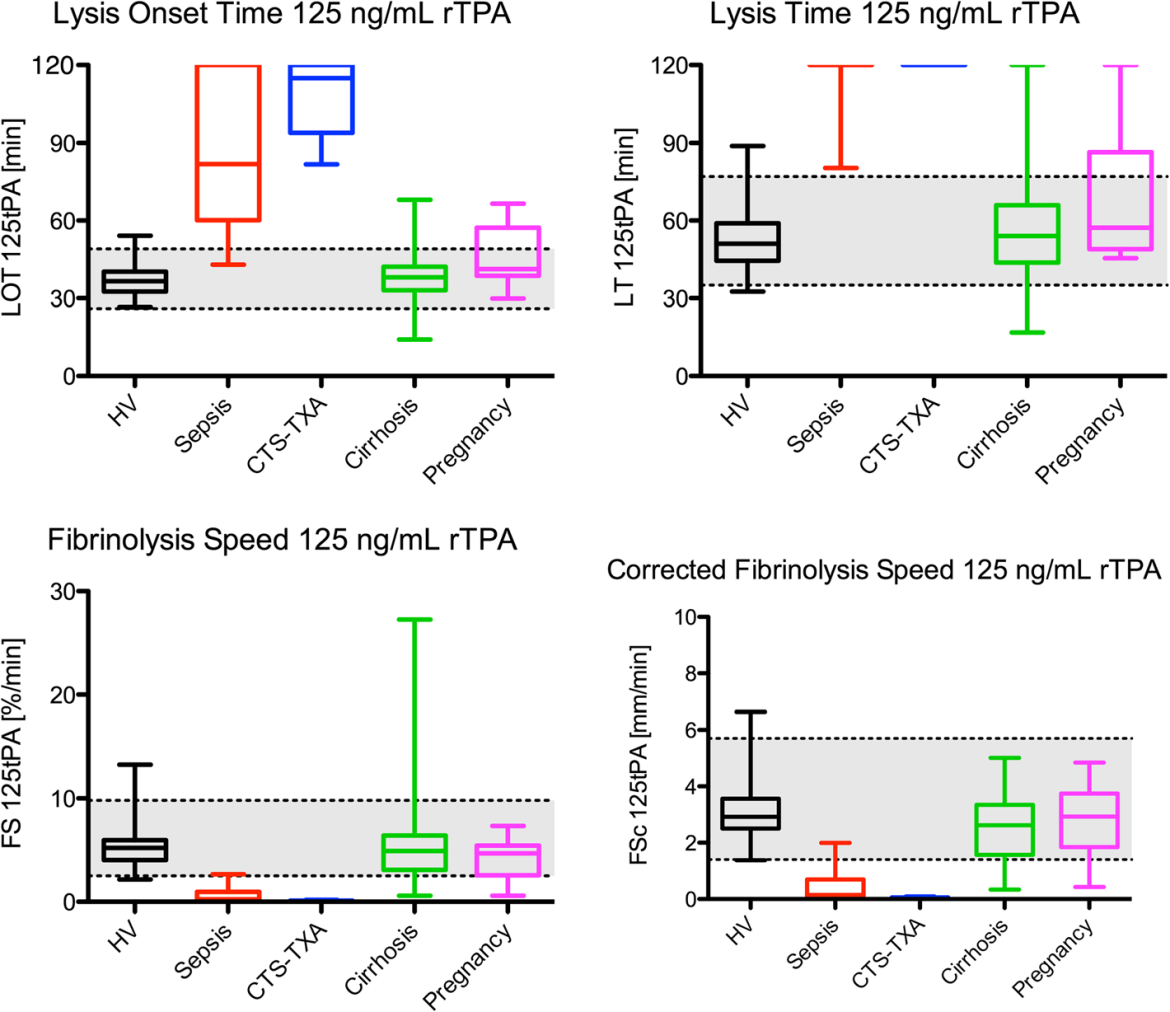
Boxplots of different ROTEM parameters at 125 ng/mL rTPA in healthy volunteers and patient groups. Sepsis and CTS are significantly different to HV (*p* < 0.001) for all four parameters. The grey area depicts the 95 % reference ranges as was calculated using the healthy volunteer data from Table 1. *CTS-TXA* cardiothoracic surgery patients using tranexamic acid, *HV* healthy volunteers, *rTPA* recombinant tissue plasminogen activator, *CT* clotting time, *MCF* maximum clot firmness, *LOT* lysis onset time, *LT* lysis time, *FS* fibrinolysis speed, defined as the decline in %/min between the LOT and LT points thus: FS = 75/(LT_min_-LOT_min_); FS_c_, corrected fibrinolysis speed in mm/min between the LOT and LT points thus: FS_c_ = (amplitude_LOT_-amplitude_LT_)/(LT_min_-LOT_min_)

At 125 ng/mL rTPA the LOT was prolonged in sepsis and CTS (*p* < 0.001), whereas the LOT was equal in cirrhosis and pregnancy. The same was seen for the LT, FS, and FS_c_ results, noting that LT could not be reached in any of the CTS patients as can be appreciated from Fig. 3.

**Fig. 4:**
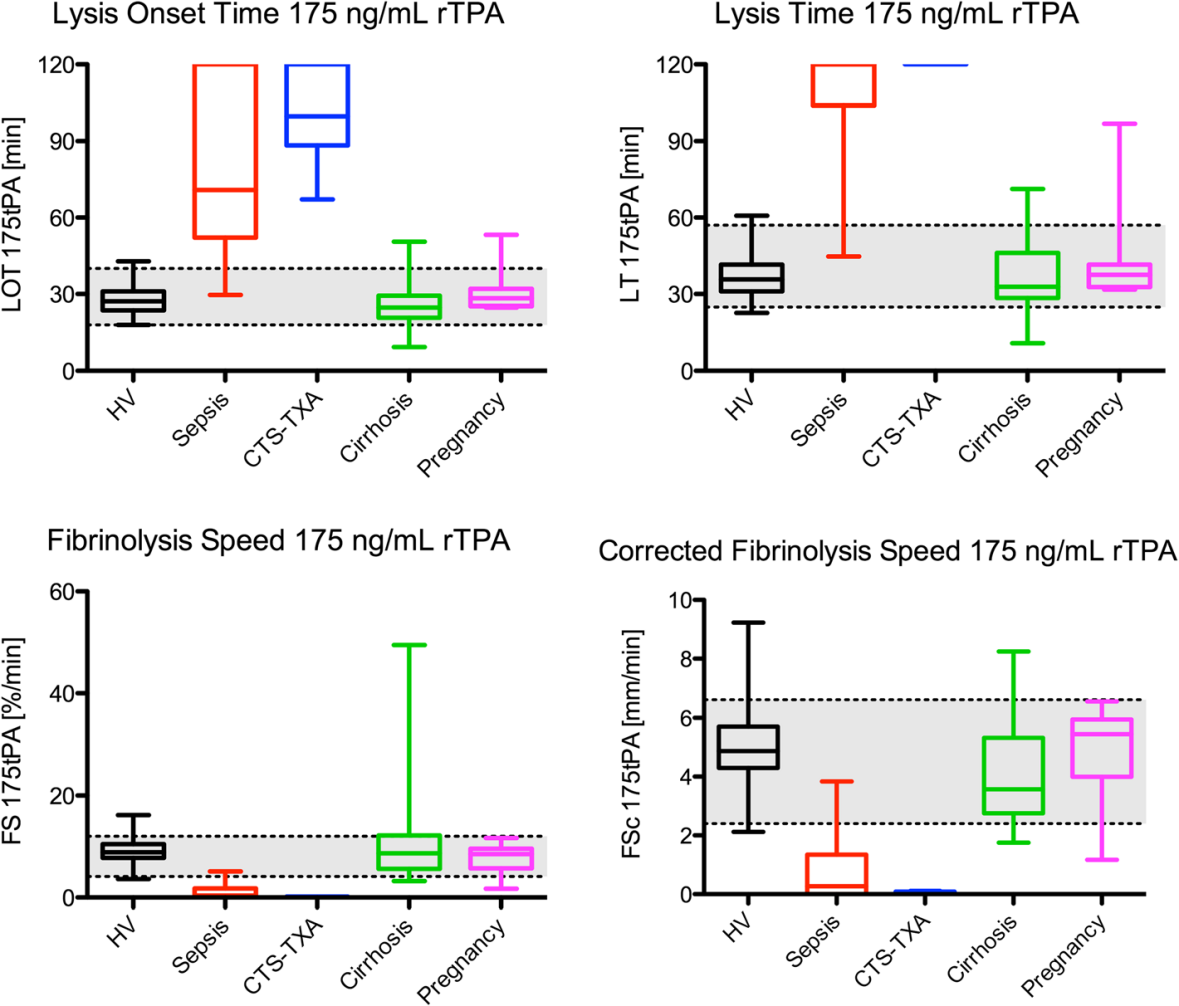
Boxplots of different ROTEM parameters at 175 ng/mL rTPA in healthy volunteers and patient groups. Sepsis and CTS are significantly different to HV (*p* < 0.001) for all four parameters. The grey area depicts the 95 % reference ranges as was calculated using the healthy volunteer data from Table 1. CTS-TXA; cardiothoracic surgery patients using tranexamic acid; HV, healthy volunteers; rTPA, recombinant tissue plasminogen activator; CT, clotting time; MCF, maximum clot firmness; LOT, lysis onset time; LT, lysis time; FS, fibrinolysis speed, defined as the decline in %/min between the LOT and LT points; thus: FS = 75/(LT_min_-LOT_min_); FS_c_, corrected fibrinolysis speed in mm/min between the LOT and LT points; thus: FS_c_ = (amplitude_LOT_-amplitude_LT_)/(LT_min_-LOT_min_)

In Fig. 4, boxplots are shown for 175 ng/mL rTPA for which the results were similar to the 125 ng/mL condition with LOT, LT, FS, and FS_c_ prolonged in sepsis and CTS (*p* < 0.001) and equal in cirrhosis and pregnancy.

**Fig. 5:**
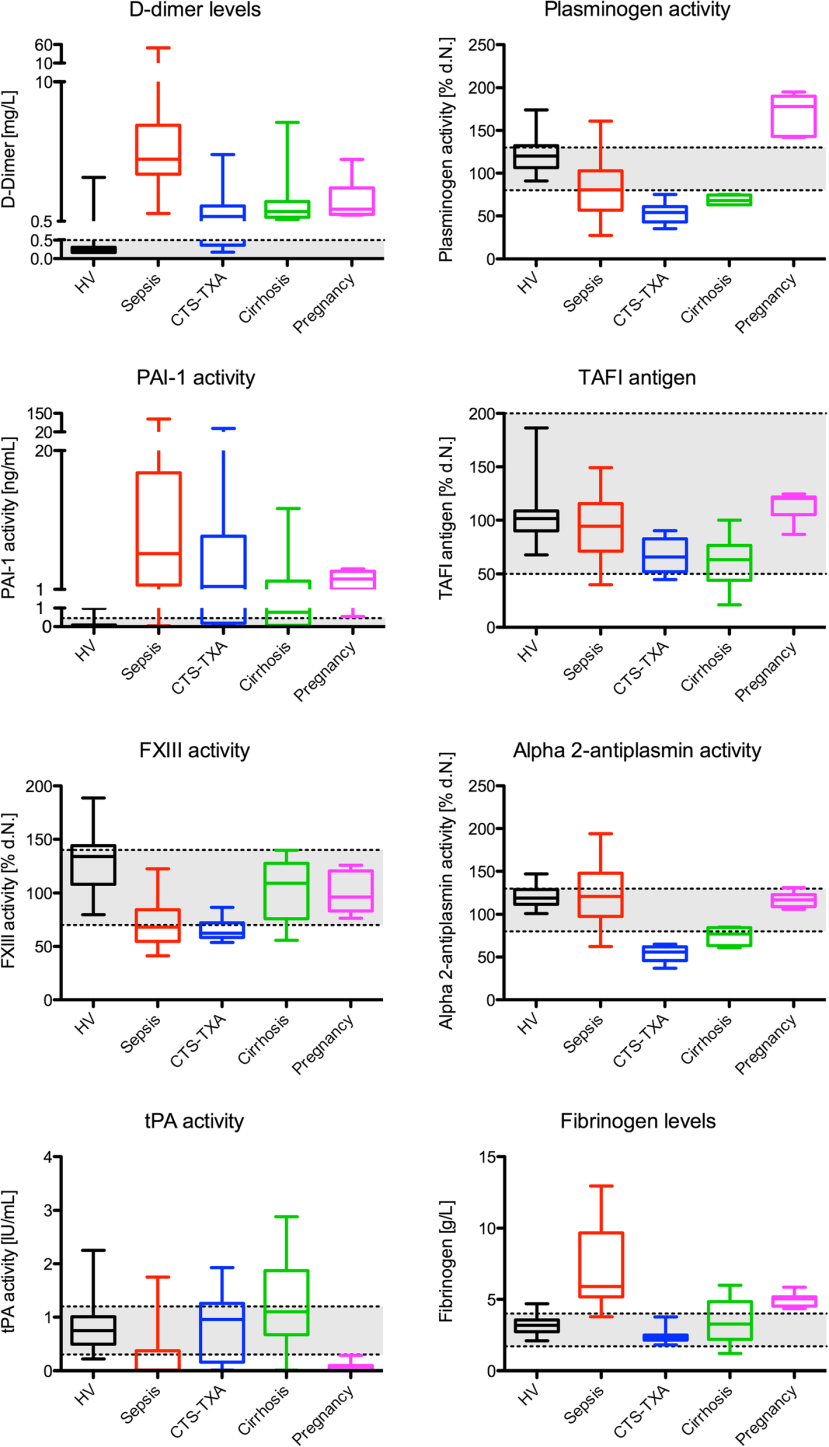
Boxplots of the fibrinolysis determinants and net effect (D-dimer) respectively, in healthy volunteers and patient groups. The grey areas depict the in house established reference range of fibrinogen and d-dimer levels, while they represent the reference ranges according to the insert of the manufacturer in the four other parameters. *CTS-TXA* cardiothoracic surgery patients using tranexamic acid, *HV* healthy volunteers, *rTPA* recombinant tissue plasminogen activator, *PAI-1* plasminogen activator inhibitor, *TAFI* thrombin activated fibrinolysis inhibitor

Determinants of the cloth strength and fibrinolytic system were assessed for each patient group and the healthy volunteers and are presented in Fig. 5.

Median d-dimer levels were elevated (>500 ng/L) in all patient groups, while median FXIII activity levels were below the reference ranges in sepsis (68.1 %) and CTS (62.3 %) only. Median plasminogen activity levels were reduced in sepsis (81 %) and especially in CTS (54 %) and cirrhosis (68 %), while being elevated in a pregnant state (178 %) when compared to healthy volunteers (119 %). The alpha 2-antiplasmin levels were equal in pregnancy (117 %) and sepsis (121 %) compared to healthy volunteers (119 %), but reduced in CTS (56 %) and cirrhosis (77 %). PAI-1 activity levels were high in all patient groups. In those with sepsis the median PAI-1 level was 6.0 ng/mL (IQR 1.7–17.0) and in those with pregnancy the median level was 2.5 ng/mL (IQR 1.1–3.6). tPA activity was low in sepsis (0.01 IU/mL) and pregnancy (0.07 IU/mL) and normal in CTS (0.96 IU/mL) and cirrhosis (1.10 IU/mL) patients. Median TAFI antigen levels were within normal range (50–200 %) in all groups. Fibrinogen, as more a procoagulant marker, together with platelet levels both account for the MCF in the 0 ng/mL rTPA condition with a significant Pearson correlation R of 0.71 and 0.57, respectively.

**Fig. 6:**
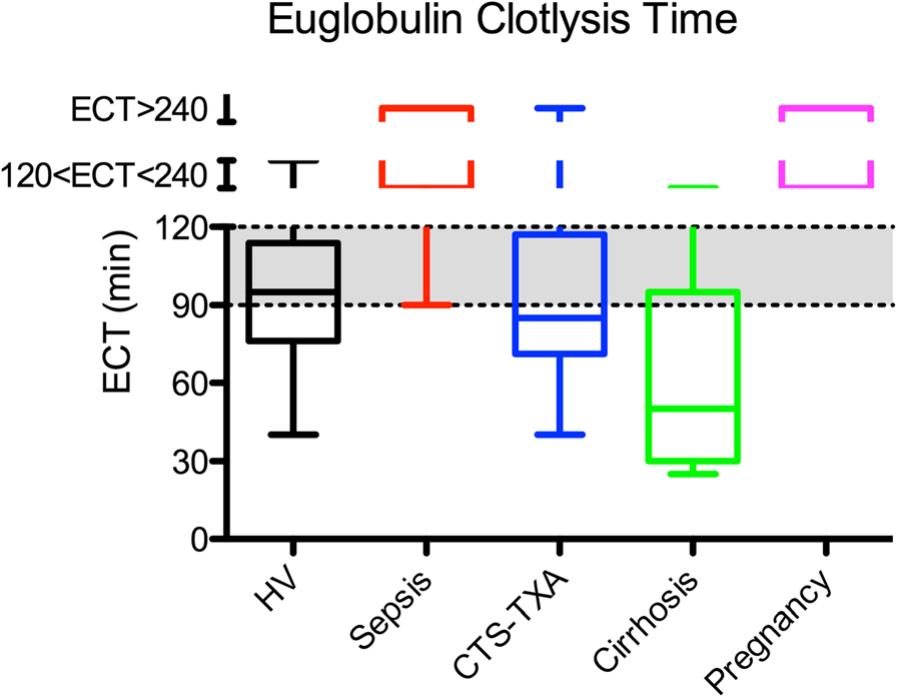
Boxplots of euglobulin clotlysis times in healthy volunteers and patient groups. The grey areas depict the reference range of euglobulin clotlysis time.* CTS-TXA* cardiothoracic surgery patients using tranexamic acid, *ECT* euglobulin clotlysis time, *HV* healthy volunteers

ECLT’s (shown in Fig. 6) in healthy volunteers revealed a median ECLT of 95 min (IQR 76–114 min). In sepsis 67 % had ECLT’s ≥4 h, 19 % ≥2 h and 14 % ≤2 h. All ECLT’s in the pregnant group were ≥2 h. In cardiothoracic surgery 80 % had ECLT shorter then 2 h with a median of 85 min (IQR 71–117). In cirrhosis, only 13 % had ECLT ≥2 h. All the others had a median of 50 min (IQR 30–95).

## Discussion

We established a protocol for the ROTEM based rTPA induced fibrinolysis assay by defining the final concentrations for TF, CaCl_2_ and rTPA using whole blood of healthy volunteers and validated this assay, to investigate, in patient cohorts their different fibrinolytic states.

The baseline assay, applying only TF and CaCl_2_ with no rTPA in whole blood, showed good reproducibility with low CV percentages similar to those reported for the commercial EXTEM test [25], with the exception of higher CV percentages for CT values, which can be attributed to the manual pipetting process used in our protocol. Addition of rTPA resulted in higher CV percentages for lysis parameters, but these were acceptable at values below 20 %. Besides, the final concentrations of rTPA of 125 and 175 ng/mL gave practical runtimes below 60 min in healthy volunteers, which is advantageous over plasma based fibrinolysis tests which include centrifugation steps. One other finding is the prolongation of the CT and attenuation of the MCF by the addition of rTPA, which is possibly due to the earlier start of fibrinolysis while the cloth is being still formed. The need for manual pipetting introduced some possible artifacts in the CT results for this study. These results are therefore operator dependable. For example we found that the CT results in male were shorter in comparison to female volunteers. This is in contrast to other research [26], but could be explained by the fact that manual pipetting was used.

When comparing to previous research, different concentrations of rTPA were chosen ranging from 50 ng/ml [10] to 100 ng/ml [11] and from 100 U/ml [21] to 1000 U/ml [20] depending on the needs for the test. The combination of a “low” and a “high” rTPA concentration would provide suitable test conditions to reflect changes in a fibrinolysis balance that might be tilted towards hypo- or hyperfibrinolysis. One could argue that the 125 ng/mL rTPA concentration may be too high already for diagnosing a hyperfibrinolytical state and that too much contrast is lost. Choosing a lower concentration of rTPA, however, resulted in longer runtimes (data not shown), hence 125 ng/mL rTPA together with a runtime of 60 min seemed optimal in normal individuals. The 175 ng/ml rTPA concentration was chosen as it diminished the lysis time by 25 % in normal healthy individuals. However, in our patient subsets still little effects were seen in sepsis and after cardiothoracic surgery at 175 ng/ml rTPA, however this concentration seems reasonable for diagnosing hypofibrinolysis. The 125 ng/ml concentration is proven to be too high for hyperfibrinolytical states. A lower concentration could improve hyperfibrinolysis testing by sacrificing short runtimes. One point which needs to be made is that for TEG (trombelastography) analysis, the use of a native TEG (non-activated) versus a RapidTEG (kaolin activated) seems to influence fibrinolysis results [27]. Whether this is also the case for our rTPA induced tissue factor activated ROTEM analysis is unclear.

When determining the effects of processing time from blood sampling to ROTEM analysis, there appears to be little effect on test outcomes within a 4-h period, which is reasonable for clinical purposes. The 95 % reference intervals were assessed using whole blood from 40 healthy volunteers. We decided however to normalize the results and calculate 95 % intervals and then transforming them back, making the 95 % reference intervals smaller than advised in order to correct for possible outliers [28]. The reference intervals in our 0 ng/mL rTPA for CT and MCF are in line with the commercial reagents for the EXTEM test. The reference intervals for the commercial EXTEM CT and MCF are 36–60s and 48–72mm respectively, as were established in our hospital healthy volunteer population. Reference intervals for LOT and LT using the EXTEM test were never established, as these seem irrelevant in a healthy population. LOT, LT, and other lysis index parameters (LI30, LI45, LI60) are used in clinical practice in overt hyperfibrinolysis.

For the clinical validation we selected patients, which according to literature could have a hyper- or hypofibrinolytic potential and compared them to healthy volunteers. The need for an rTPA induced method of assessing fibrinolysis in the ROTEM assay becomes obvious when comparing test conditions with rTPA versus those without rTPA addition. Within a time span of 2 h no distinction can be made between any of the groups and healthy population with regard to fibrinolytic potential, either visually or by using CT, MCF, LOT, or LT parameters in the absence of added rTPA. As illustrated in Fig. 2, marked differences between healthy persons on the one hand and different patient populations on the other hand are detectable with added rTPA.

In subjects with sepsis but without an overt state of DIC (according to ISTH criteria) a hypofibrinolytic state (long LOT and LT combined with reduced FS and FSc) in conjunction with a marked procoagulant state (high MCF) is evident, despite the fact that high D-dimer levels suggest a high fibrinolytic activity. ECLT testing showed a hypofibrinolytic state in sepsis, which is in agreement with our rTPA induced ROTEM assay. D-dimer levels are determinants of different processes [29], including the generation of fibrin as well as the rather nonspecific cleavage of polymerized fibrin molecules by various enzymes including plasmin but also elastase, trypsin and others that are released during sepsis by neutrophils and other inflammatory cells. The high PAI-1 activity is thought to be primarily responsible for the attenuated fibrinolysis in patients with sepsis. Transient elevation of tPA and PAI-1 exists due to endothelial damage, but soon after, tPA antigen and activity is lowered through inactivation by increasing levels of PAI-1 [30]. Theoretically, upon progression of sepsis and occurrence of overt DIC alpha 2-antiplasmin would be consumed and the ROTEM pattern may change. This was not found in the early sepsis group and we did not address conversion to DIC in the present investigation.

In patients with CTS being administered tranexamic acid a hypofibrinolytic state is observed (reduced FS and FSc), although an overall reduced maximum clot firmness to start with is present in this group. The ECLT in this group did not show a hypofibrinolytic state, but rather a normal state when compared to healthy volunteers. The difference between the ECLT and our ROTEM assay appears to be due to the fact that tranexamic acid does not precipitate in the ECLT test [31]. Reduced MCF is seen following dilutional effects during ECC by lowering fibrinogen levels and platelet numbers. Furthermore, consumption of fibrinogen during cardiac surgery and the subsequent raised d-dimer levels are seen in our study. PAI-1 levels are high in our study for CTS, which, next to tranexamic acid, could strengthen clot firmness and prevent excessive breakdown by fibrinolytic enzymes (which could be reflected by the reduced activity of plasminogen) and could counteract the reduced antifibrinolytic activity of alpha 2-antiplasmin. Reduced activity of FXIIIa together with a hypofibrinolytic state is seen in CTS [32], which could be the result of FXIII consumption and dilution. The additional effect of tranexamic acid resulted in a hypofibrinolytical profile in our ROTEM assay, while in the one patient not having had tranexamic acid it showed a normal fibrinolytic profile compared to healthy volunteers. Tranexamic acid counteracts the effects of ECC induced endogenous tPA release, caused by shear stress on the vessel wall [33] and haemodilutional effects of cardiothoracic surgery [34], and may stabilize clots even more than needed.

Cirrhotic liver disease patients do not seem to differ from healthy volunteers regarding their global fibrinolytic rTPA-ROTEM profile with equal LOT, LT, FS, and FSc in both rTPA induced conditions together with a globally reduced procoagulant state. The ECLT was markedly reduced. Both findings are coherent with other studies as was researched and discussed previously by our group [35]. This reset balance in hemostasis is characterized by a reduced level of both the procoagulant (e.g. fibrinogen) and the antifibrinolytic factors (e.g. low alpha2-antiplasmin) [36]. tPA is thought to be increasing over time, while PAI-1 activity levels are decreasing [37]. This could theoretically result in more pronounced hyperfibrinolysis. High d-dimer levels indicate ongoing lysis. Again, high d-dimer levels are seen in almost any thrombotic disease state. Some individuals with cirrhosis showed very high fibrinolysis speeds (FS), but when these are corrected for their already reduced MCF, the corrected fibrinolysis speed (FSc) might give a more accurate view on fibrinolysis potential. The use of the FSc seem to be more helpful when interpreting fibrinolysis potentials instead of the FS in assessing the hemostatic balance by correcting for fibrinogen and platelet counts which are the main correlates for MCF. Again the 125 ng/ml rTPA concentration might be too high in patients with hyperfibrinolysis activity and a lower concentration of rTPA could reveal more pronounced hyperfibrinolysis in cirrhosis.

Pregnancy is associated with a gradual increase in PAI-1 over time [38], as confirmed in our study. TAFI and fibrinogen levels are also higher in comparison to healthy volunteers [39–41]. tPA activity levels were lower in comparison to the healthy volunteers, which is comparable to previous studies [41]. An incline in tPA levels was shown followed by a drop in tPA levels postpartum. In our study, reduced tPA levels could result in higher than normal plasminogen activity. ROTEM reference values in pregnancy were established previously in a study from our clinics and are in line with the present findings [42]. Hypofibrinolysis on our ROTEM assay could not be detected, but was clearly present in the ECLT test.

Fibrinogen levels and also platelet levels seem to correlate well with MCF for any given group, an all-by-all no new finding [43].

Thus far the test is able to pickup hypofibrinolysis accurately in groups of patients with the 175 ng/mL rTPA concentration and could guide therapy in CTS and sepsis. One could argue that the amount of tranexamic acid given to CTS patients is too much if even at the high concentration of rTPA no fibrinolysis is seen within 2 h. This assay could be used to titrate the drug following the ROTEM graphs and results in conjunction with clinical relevant outcomes. Trauma induced coagulopathy (TIC) is a very interesting syndrome in which overt hyperfbrinolysis is sometimes seen using ROTEM without the need for rTPA addition. As is shown by the Crash-2 trial [8] the use of tranexamic acid reduces mortality when given within 3 h of the trauma. In our study a trauma population could not be assessed because of low numbers of severe trauma patients seen in our hospital during normal operating hours. Much research is done in this study population using TEG technology [12]. ROTEM and TEG are essentially the same techniques, but differ slightly in measuring principle, reagents used, and naming conventions [43]. In a recent paper by Moore et al. [44] hyperfibrinolysis seen on non-rTPA induced TEG analysis was associated with higher mortality in comparison to a physiological subgroup, while shutdown of hyperfibrinolysis was associated with lower mortality rates in comparison to hyperfibrinolysis, but these still being higher than the physiological subgroup. This resulted in a U-shaped optimum curve in which careful titration of tranexamic acid could result in better survival rates. High mortality rate due to fibrinolysis shutdown was also found by Raza et al. [1]. A recent review by Dobson et al. [45] discusses the timeline in trauma induced coagulopathy and the review by Napolitano et al. [46] extensively researched the literature to make recommendations regarding tranexamic acid use in trauma. For this purpose implementation of our rTPA-ROTEM assay may shed more light on this subject matter.

As a limitation, hyperfibrinolysis could not be shown in a group of patients with cirrhosis when averaging their results. However, some individual patients do show a hyperfibrinolytic potential, while others do not. Still, this assay might be useful for individual patients with cirrhotic liver disease to diagnose (and treat) hyperfibrinolysis, although the rTPA addition needs titrating to a lower concentration.

Another limitation is the manual pipetting that is required up till now. Having ready to use solutions and implementing them together with the automatic pipetting function on the ROTEM device could greatly reduce the inter-observer variability, especially for the clotting times.

Our rTPA induced fibrinolysis ROTEM assay showed different results in comparison to the ECLT test. This is because there are essential differences between both tests. The ECLT is plasma based and due to acidification of the plasma only few factors are precipitated, the euglobulin fraction. As such, the role of red blood cells and thrombocytes is excluded in the ECLT on fibrinolysis regulation. This makes our test a valuable addition to the arsenal of fibrinolysis testing.

## Conclusions

In conclusion, we developed a reproducible whole blood rTPA ROTEM assay for diagnosing hypofibrinolytic potential with short turnaround times, which in healthy volunteers seems to produce acceptable variations resulting in narrow reference intervals. The 175 ng/ml rTPA concentration appears to be sensitive for the medication effect of tranexamic acid on fibrinolysis, which is in contrast to the ECLT for tranexamic acid. This 175 ng/ml concentration may distinguish a hypo- from a normofibrinolytic potential. The implementation of this test needs to be evaluated in more prominent hyperfibrinolytic disease states, like trauma or thrombolysis therapy, in order to address the potential utility in the diagnostic workup of patients with fibrinolytic dysfunction, as well as in guiding anti-fibrinolytic management.

## References

[1] Raza I, Davenport R, Rourke C, Platton S, Manson J, Spoors C (2013). The incidence and magnitude of fibrinolytic activation in trauma patients. J Thromb Haemost.

[2] Rijken DC, Kock EL, Guimaraes AH, Talens S, Darwish Murad S, Janssen HL (2012). Evidence for an enhanced fibrinolytic capacity in cirrhosis as measured with two different global fibrinolysis tests. J Thromb Haemost.

[3] Smith AA, Jacobson LJ, Miller BI, Hathaway WE, Manco-Johnson MJ (2003). A new euglobulin clot lysis assay for global fibrinolysis. Thromb Res.

[4] Wada H (2004). Disseminated intravascular coagulation. Clin Chim Acta.

[5] ten Cate H (2000). Pathophysiology of disseminated intravascular coagulation in sepsis. Crit Care Med.

[6] Zeerleder S, Schroeder V, Lammle B, Wuillemin WA, Hack CE, Kohler HP (2007). Factor XIII in severe sepsis and septic shock. Thromb Res.

[7] McCormack PL (2012). Tranexamic acid: a review of its use in the treatment of hyperfibrinolysis. Drugs.

[8] Roberts I, Shakur H, Afolabi A, Brohi K, Coats T, Dewan Y (2011). The importance of early treatment with tranexamic acid in bleeding trauma patients: an exploratory analysis of the CRASH-2 randomised controlled trial. Lancet.

[9] Chitlur M (2012). Challenges in the laboratory analyses of bleeding disorders. Thromb Res.

[10] Kupesiz A, Rajpurkar M, Warrier I, Hollon W, Tosun O, Lusher J (2010). Tissue plasminogen activator induced fibrinolysis: standardization of method using thromboelastography. Blood Coagul Fibrinolysis.

[11] Dirkmann D, Radu-Berlemann J, Gorlinger K, Peters J (2013). Recombinant tissue-type plasminogen activator-evoked hyperfibrinolysis is enhanced by acidosis and inhibited by hypothermia but still can be blocked by tranexamic acid. J Trauma Acute Care Surg.

[12] Moore HB, Moore EE, Chapman MP, Gonzalez E, Slaughter AL, Morton AP (2015). Viscoelastic measurements of platelet function, not fibrinogen function, predicts sensitivity to tissue-type plasminogen activator in trauma patients. J Thromb Haemost.

[13] Gallimore MJ, Harris SL, Tappenden KA, Winter M, Jones DW (2005). Urokinase induced fibrinolysis in thromboelastography: a model for studying fibrinolysis and coagulation in whole blood. J Thromb Haemost.

[14] Dargaud Y, Prevost C, Lienhart A, Claude Bordet J, Negrier C (2011). Evaluation of the overall haemostatic effect of recombinant factor VIIa by measuring thrombin generation and stability of fibrin clots. Haemophilia.

[15] Franz RC (2009). ROTEM analysis: a significant advance in the field of rotational thrombelastography. S Afr J Surg.

[16] Mittermayr M, Streif W, Haas T, Fries D, Velik-Salchner C, Klingler A (2008). Effects of colloid and crystalloid solutions on endogenous activation of fibrinolysis and resistance of polymerized fibrin to recombinant tissue plasminogen activator added ex vivo. Br J Anaesth.

[17] Katori N, Tanaka KA, Szlam F, Levy JH (2005). The effects of platelet count on clot retraction and tissue plasminogen activator-induced fibrinolysis on thrombelastography. Anesth Analg.

[18] Shenkman B, Livnat T, Budnik I, Tamarin I, Einav Y, Martinowitz U (2012). Plasma tissue-type plasminogen activator increases fibrinolytic activity of exogenous urokinase-type plasminogen activator. Blood Coagul Fibrinolysis.

[19] Viuff D, Andersen S, Sorensen BB, Lethagen S (2010). Optimizing thrombelastography (TEG) assay conditions to monitor rFVIIa (NovoSeven) therapy in haemophilia A patients. Thromb Res.

[20] Nielsen VG, Cankovic L, Steenwyk BL (2007). Epsilon-aminocaproic acid inhibition of fibrinolysis in vitro: should the ‘therapeutic’ concentration be reconsidered?. Blood Coagul Fibrinolysis.

[21] Nielsen VG, Cohen BM, Cohen E (2006). Elastic modulus-based thrombelastographic quantification of plasma clot fibrinolysis with progressive plasminogen activation. Blood Coagul Fibrinolysis.

[22] Annane D, Bellissant E, Cavaillon JM (2005). Septic shock. Lancet.

[23] Toh CH, Downey C (2005). Performance and prognostic importance of a new clinical and laboratory scoring system for identifying non-overt disseminated intravascular coagulation. Blood Coagul Fibrinolysis.

[24] Kowalski E, Kopec M (1959). Niewiarowski. An evaluation of the euglobulin method for the determination of fibrinolysis. J Clin Pathol.

[25] Kitchen DP, Kitchen S, Jennings I, Woods T, Walker I (2010). Quality assurance and quality control of thrombelastography and rotational Thromboelastometry: the UK NEQAS for blood coagulation experience. Semin Thromb Hemost.

[26] Sucker C, Tharra K, Litmathe J, Scharf RE, Zotz RB (2011). Rotation thromboelastography (ROTEM) parameters are influenced by age, gender, and oral contraception. Perfusion.

[27] Thai J, Reynolds EJ, Natalia N, Cornelissen C, Lemmens HJ, Hill CC (2011). Comparison between RapidTEG(R) and conventional thromboelastography in cardiac surgery patients. Br J Anaesth.

[28] Horn PS, Pesce AJ (2003). Reference intervals: an update. Clin Chim Acta.

[29] Kabrhel C, Mark Courtney D, Camargo CA, Plewa MC, Nordenholz KE, Moore CL (2010). Factors associated with positive D-dimer results in patients evaluated for pulmonary embolism. Acad Emerg Med.

[30] Gando S (2013). Role of fibrinolysis in sepsis. Semin Thromb Hemost.

[31] Katz J, Lurie A, Becker D, Metz J (1970). The euglobulin lysis time test: an ineffectual monitor of the therapeutic inhibition of fibrinolysis. J Clin Pathol.

[32] Chandler WL, Patel MA, Gravelle L, Soltow LO, Lewis K, Bishop PD (2001). Factor XIIIA and clot strength after cardiopulmonary bypass. Blood Coagul Fibrinolysis.

[33] Yavari M, Becker RC (2009). Coagulation and fibrinolytic protein kinetics in cardiopulmonary bypass. J Thromb Thrombolysis.

[34] Woodman RC, Harker LA (1990). Bleeding complications associated with cardiopulmonary bypass. Blood.

[35] Kleinegris MC, Bos MH, Roest M, Henskens Y, Ten Cate-Hoek A, Van Deursen C (2014). Cirrhosis patients have a coagulopathy that is associated with decreased clot formation capacity. J Thromb Haemost.

[36] Tripodi A, Mannucci PM (2011). The coagulopathy of chronic liver disease. N Engl J Med.

[37] Ferguson JW, Helmy A, Ludlam C, Webb DJ, Hayes PC, Newby DC (2008). Hyperfibrinolysis in alcoholic cirrhosis: relative plasminogen activator inhibitor type 1 deficiency. Thromb Res.

[38] Robb AO, Mills NL, Din JN, Cameron S, Ludlam CA, Newby DE (2009). Acute endothelial tissue plasminogen activator release in pregnancy. J Thromb Haemost.

[39] Knol HM, Veeger NJ, Middeldorp S, Hamulyak K, Van Der Meer J (2009). High thrombin-activatable fibrinolysis inhibitor levels may protect against recurrent fetal loss. J Thromb Haemost.

[40] Mousa HA, Downey C, Alfirevic Z, Toh CH (2004). Thrombin activatable fibrinolysis inhibitor and its fibrinolytic effect in normal pregnancy. Thromb Haemost.

[41] Cerneca F, Ricci G, Simeone R, Malisano M, Alberico S, Guaschino S (1997). Coagulation and fibrinolysis changes in normal pregnancy. Increased levels of procoagulants and reduced levels of inhibitors during pregnancy induce a hypercoagulable state, combined with a reactive fibrinolysis. Eur J Obstet Gynecol Reprod Biol.

[42] de Lange NM, van Rheenen-Flach LE, Lance MD, Mooyman L, Woiski M, van Pampus EC, et al. Peri-partum reference ranges for ROTEM(R) thromboelastometry. Br J Anaesth. 2014. 10.1093/bja/aet480 24486836

[43] Luddington RJ (2005). Thrombelastography/thromboelastometry. Clin Lab Haematol.

[44] Moore HB, Moore EE, Gonzalez E, Chapman MP, Chin TL, Silliman CC (2014). Hyperfibrinolysis, physiologic fibrinolysis, and fibrinolysis shutdown: the spectrum of postinjury fibrinolysis and relevance to antifibrinolytic therapy. J Trauma Acute Care Surg.

[45] Dobson GP, Letson HL, Sharma R, Sheppard FR, Cap AP (2015). Mechanisms of early trauma-induced coagulopathy: The clot thickens or not?. J Trauma Acute Care Surg.

[46] Napolitano LM, Cohen MJ, Cotton BA, Schreiber MA, Moore EE (2013). Tranexamic acid in trauma: how should we use it?. J Trauma Acute Care Surg.

